# Variation between the oral and faecal microbiota in a free-living passerine bird, the great tit (*Parus major*)

**DOI:** 10.1371/journal.pone.0179945

**Published:** 2017-06-29

**Authors:** Lucie Kropáčková, Hana Pechmanová, Michal Vinkler, Jana Svobodová, Hana Velová, Martin Těšičký, Jean-François Martin, Jakub Kreisinger

**Affiliations:** 1 Department of Zoology, Faculty of Science, Charles University, Prague, Czech Republic; 2 Department of Ecology, Faculty of Environmental Science, Czech University of Life Science Prague, Prague, Czech Republic; 3 Montpellier-SupAgro, UMR CBGP, Montferrier-sur-Lez, France; Universita degli Studi di Milano-Bicocca, ITALY

## Abstract

The gastrointestinal tract of vertebrates is inhabited by diverse bacterial communities that induce marked effects on the host physiology and health status. The composition of the gastrointestinal microbiota is characterized by pronounced taxonomic and functional variability among different regions of the vertebrate gastrointestinal tract. Despite the relatively solid knowledge on the among-region variations of the gastrointestinal microbiota in model mammalian species, there are only a few studies concerning among-region variations of the gastrointestinal microbiota in free-living non-mammalian vertebrate taxa. We used Illumina MiSeq sequencing of bacterial 16S rRNA amplicons to compare the diversity as well as taxonomic composition of bacterial communities in proximal vs. distal parts of the gastrointestinal tract (represented by oral swabs and faecal samples, respectively) in a wild passerine bird, the great tit (*Parus major*). The diversity of the oral microbiota was significantly higher compared to the faecal microbiota, whereas interindividual variation was higher in faecal than in oral samples. We also observed a pronounced difference in taxonomic content between the oral and faecal microbiota. Bacteria belonging to the phyla Proteobacteria, Firmicutes and Actinobacteria typically dominated in both oral and faecal samples. A high abundance of bacteria belonging to Tenericutes was observed only in faecal samples. Surprisingly, we found only a slight correlation between the faecal and oral microbiota at the within-individual level, suggesting that the microbial composition in these body sites is shaped by independent regulatory processes. Given the independence of these two communities at the individual level, we propose that simultaneous sampling of the faecal and oral microbiota will extend our understanding of host vs. microbiota interactions in wild populations.

## Introduction

Animal bodies are inhabited by taxonomically and functionally diverse communities of bacteria [1–3] that modulate their host's physiology and health status [4,5]. The modulations mediated by microbial communities are believed to be beneficial in most cases. However, adverse effects can be elicited by obligatory pathogenic bacterial species invading host bodies or facultative pathogens dwelling in immunocompromised hosts [6–8].

The host-associated microbiota exhibits pronounced spatial variation among different parts of the animal body [1,9–11]. Out of this complex system of host-associated microbial consortia, the microbiota of the gastrointestinal tract (hereafter GIT) has attracted considerable research attention during the past two decades. The GIT microbiota provides important benefits to the host, including increased efficiency of food digestion [12–14], stimulation of the immune system [15,16], defence against pathogens [17,18] and beneficial effects on the development and functioning of the gut and central nervous system [19,20]. At the same time, GIT microbiota dysbiosis is associated with metabolic, autoimmune and neurological disorders [21–24]. Last but not least, many bacterial pathogens invade animal bodies through the GIT [25–27], providing a further argument for the importance of the GIT microbiota on animal fitness.

Most current studies focusing on the GIT microbiota, use the microbiota of faecal samples, putatively representing the GIT microbiota of the lower intestine, as a proxy [3,28–30]. However, there is compelling evidence that microbial composition exhibits marked variation between different compartments of the GIT [9,31–33]. Such variation occurs due to spatial changes in biotic and abiotic factors among different GIT regions, including acidity levels, concentrations of oxygen, cholic acids and nutrients as well as changes in the host’s immunity [34–36]. As the relative contributions of these mechanisms to microbial structure may be independent both at the between-GIT region level and the interindividual level, the resulting within-individual correlation of the microbial content among different GIT regions can be of rather low effect size (Kreisinger et al., in prep, [37]). At the same time, the microbiota in different GIT regions can be associated with distinct effects on the hosts' phenotype [33,38]. Consequently, studies relying on samples from single GIT regions, or those based solely on faecal samples, can provide only a limited view on the outcome of interactions between the GIT microbiota and the host.

A few studies have analysed host vs. microbiota interactions using microbiota samples from multiple GIT regions [9,33,37]. However, as acquiring the corresponding samples was typically performed in a destructive way, this approach does not enable assessments of temporal variation of the GIT microbiota based on longitudinal sampling of the same individual. Furthermore, destructive sampling might be ethically questionable in the case of research on protected wild species. Although biopsies taken from different GIT regions offer a non-destructive alternative [39–41], such an approach is methodically challenging, which limits its broader applicability. Therefore, an application of alternative sampling protocols that are non-destructive but allow sampling of multiple GIT regions is desirable.

Moreover, most current knowledge on microbial variation along the GIT relies on data from mammalian captive-bred models and humans [40–42]. Patterns of spatial variation of the microbiota along the GIT in wild populations and in non-mammalian taxa are still understudied, however [9,31,32,43]. This sort of knowledge is crucial for understanding host vs. microbiota interactions, since previous studies, mostly based on samples from single GIT regions, have revealed that the taxonomic and functional content of the GIT microbiota varies considerably between captive-bred and wild populations of the same species [44–47], as well as between mammals vs. other vertebrates [28,30,48,49]. Consequently, approaches based on sampling of multiple GIT regions are essential for understanding host vs. microbiota interactions in wild populations, including resulting effects on the host's fitness.

Here we applied high-throughput amplicon sequencing of bacterial 16S rRNA to study the variation in microbiota among samples of proximal and distal parts of the GIT (represented by oral swabs and faecal samples) noninvasively collected in a free-living passerine bird. According to research on mammals, microbial communities of both the proximal and distal GIT are shaped to a large extent by host-intrinsic regulatory mechanisms, while the effect of environmental bacteria on the composition of these communities is usually limited [43,50]. At the same time, however, host-specific factors affecting microbial populations differ between the proximal and distal GIT in mammals. Diet composition, infection by intestinal parasites and genetic factors are crucial factors leading to lower GIT microbiota variation [51–53]. On the other hand, specific properties of the saliva and gingival crevicular fluid, and to a lesser extent diet, have important effects on the proximal GIT microbiota [54]. Importantly, however, distinct mechanisms seem to drive the variation of host-associated microbiota in mammals vs. non-mammalian vertebrates [28,30,48,55].

In this study, great tit (*Parus major*) was selected as a model species. The great tit is an eminent model species for the functionally and evolutionary oriented branches of ecological research [56,57]. Despite the current interest in the emerging topic of host vs. GIT microbiota interactions [58–61], there is still rather limited knowledge on these interactions in the great tit [62]. Importantly, no previous studies on the great tit have used culture-independent high-throughput sequencing to characterize the microbial communities associated with this host. In addition, to our knowledge, variation in the microbiota colonizing different parts of body of this species as well as other passerines has not yet been addressed. We compared the diversity, interindividual variation and taxonomic composition of the microbiota from the proximal vs. distal parts of the GIT. Finally, we tested if there was any correlation between the proximal and distal GIT microbiota at the within-individual level.

## Materials and methods

### Field sampling

Faecal and oral samples used in this study were collected from putatively unrelated adult individuals (n = 29) of the great tit population breeding in artificial nest boxes in the Ďáblický háj forest (50°08'12.4"N, 14°27'57.2"E, Prague, Czech Republic). The sampling locality is covered by secondary deciduous forest with a minor admixture of coniferous trees. All samples were obtained within one week in mid-May 2014.

Collection of microbial samples was performed as follows: adult individuals were captured in mist nets and placed in clean paper bags for approx. 15–20 minutes. Samples of faeces were subsequently collected from the paper surface. The oral microbiota was sampled using sterile microbiological nylon swabs (minitip FLOQSwabs, Copan, Italy) by wiping the oral cavity and upper side of the beak. Both faecal and oral samples were immediately placed in sterile DNA/RNA free cryotubes (Simport, Canada) filled with a self-made DNA/RNA-stabilising buffer on the basis of RNA later (protocol available upon request) and transferred to -80°C within two days. The sex of sampled individuals was determined by external phenotypic traits (e.g. [63]). Data on the body mass and tarsus length were used for calculation of the scaled body mass index following Peig and Green [64]. Birds were then individually marked using aluminium rings following the regulations of the Czech Bird Ringing Centre and released.

All field procedures were approved by the ethical committee of the Czech Academy of Sciences (107/2009).

### Microbial genotyping

Metagenomic DNA from faecal and oral samples was extracted in a laminar flow cabinet using the PowerSoil DNA isolation kit (MO BIO Laboratories Inc., USA). To optimise the efficiency of DNA isolation, samples were homogenised using a MagnaLyzer (Roche, Switzerland) for 30s at 6000rpm and extracted DNA was eluted in 50 μl of elution buffer. From 29 sampled individuals, DNA isolated from oral and faecal samples was not of sufficient quantity in 9 and 12 cases, respectively, and therefore these samples were not included in further analyses. The final dataset thus included 17 faecal and 20 oral samples (S1 Table).

Following the recommendations of Klindworth et al., [65], primers covering the V3-V4 variable region of bacterial 16S rRNA (i.e. S-D-Bact-0341-b-S-17 [CCTACGGGNGGCWGCAG] and S-D-Bact-0785-a-A-21 [GACTACHVGGGTATCTAATCC]) were used during the PCR step. Both forward and reverse primers were tagged with 10bp barcodes designed by TagGD software [66]. For the polymerase chain reaction (PCR) we used 8 μl of KAPA HIFI Hot Start Ready Mix (Kapa Biosystems, USA), 0.37 μM of each primer and 7 μl of DNA template. PCR conditions were as follows: initial denaturation at 95°C for 5 min, followed by 35 cycles each of 98°C (20 sec), 61°C (15 sec) and 72°C (40 sec), and a final extension at 72°C (5 min). For individual samples, we prepared technical PCR duplicates. The PCR products, together with negative controls (PCR products of blank DNA isolates), were run on a 1.5% agarose gel and concentration of the PCR product was assessed based on gel band intensity using GenoSoft software (VWR International, Belgium). Samples were subsequently pooled at equimolar concentration. As we did not observe any visible PCR products in negative controls, therefore this type of samples was not included into the final pool. The pooled samples then were run on another 1.5% agarose gel, with bands of appropriate size excised from the gel and purified using the High Pure PCR product Purification Kit (Roche, Switzerland) according to the manufacturer’s instructions. Sequencing adaptors were ligated using TruSeq nano DNA library preparation kits (Illumina, USA) and the resulting amplicon libraries sequenced on a single Miseq run (Illumina, USA) using v3 chemistry and 2 × 300 bp paired-end reads. Raw sequencing data are avialable at http://www.ebi.ac.uk/ena/data/view/PRJEB19204 and sample metadata in S1 Table.

### Bioinformatic processing of 16S rRNA data

Paired-end Illumina reads were merged using PEAR [67], and de-mutiplexed using mothur [68] and custom R/Bioconductor scripts (available from the authors on request). We then used the Lotus pipeline [69] for quality filtering of the FASTQ files. Sequences were excluded if the average quality score was lower than 30 or if the average quality score within a 50 bp sliding window decreased below 25. UCHIME (implemented in the Lotus pipeline) [70] was used alongside the gold.fna database (available at http://sourceforge.net/projects/microbiomeutil/files) for the detection and elimination of chimeric sequences. The resulting 16S rRNA sequences were clustered at a 97% similarity threshold using UPARSE [71] in order to define Operational Taxonomic Units (OTU). Taxonomic assignation of representative sequences for each OTU was performed using the RDP classifier [72] and the GreenGenes reference database, (version gg_13_5) [73]. Representative sequences were further aligned using PyNAST [74], the maximum likelihood tree being constructed using FastTree [75]. We observed a considerable excess of chloroplast sequences in our dataset (17.7%). Chloroplast OTUs together with OTUs that were not assigned to any bacterial phylum were considered as diet contaminants or sequencing artefacts, respectively, and we excluded them from all downstream analyses. The resulting OTU tables, sample metadata, OTU tree and taxonomic annotations for individual OTUs were merged into a phyloseq object [76] for statistical analysis in R version 3.2.3 [77].

### Statistical analyses

In order to account for uneven sequencing depth among samples, statistical analyses were calculated based on the rarefied OTU table unless otherwise stated. The number of observed OTUs, Shannon diversity and Chao1 based predictions of total microbial diversity for individual samples were calculated using phyloseq [76]. Linear Mixed Effect models (LME, package lme4) [78] were used to test differences in diversity between faecal vs. oral microbiota. To account for statistical nonindependence, the effects of an individual were included as a random intercept. In addition, analysis of variance (ANOVA), running separately on samples from each GIT region, was applied to test differences in microbial alpha diversity between males vs. females and due to scaled body mass index.

We further used Principal Coordinate Analysis (PCoA) based on Bray-Curtis, Jaccard, weighted and unweighted UniFrac [79] distances between samples to visualize the contrast in the composition between faecal and oral microbiota. Adonis (i.e. analysis of variance based on distance matrices) was applied to assess the statistical significance and proportion of variance explained by the contrast in microbial composition between faecal and oral samples. Individual identity was included as a constraint for permutations (i.e. “strata”) in adonis models to account for data nonindependence. Betadisper was further applied to test for the difference in interindividual variation of microbial composition between the two GIT regions. The effects of sex and scaled body mass index were assessed via adonis analyses running separately on faecal and oral samples. For individuals where both oral and faecal microbiota were analysed (n = 8), we used Pearson correlations to assess if there was any interrelationship in microbial alpha diversity between the two GIT regions. Next, within-individual correlations of the microbial composition between oral vs. faecal samples was assessed via Mantel's test. Finally, using Spearman's correlations, we tested if relative abundances of OTUs were correlated between the two GIT regions. This analysis was run on a subset of 216 OTUs that were present both in the oral and faecal microbiota of those individuals for which both these samples were available.

The LME-based approach was further used to identify OTUs whose abundances differed between oral and faecal samples. These analyses were performed on a subset of 240 OTUs (comprising 90.5% of all high-quality reads) that were detected in at least five samples. For each OTU-specific LME, Box-Cox transformed read counts were used as a response, whereas the effect of GIT regions and individual identity were included as the explanatory variable and random intercept, respectively. In addition, Box-Cox transformed total number of reads per individual samples was included as an offset in LMEs (i.e. assuming its direct relationship with the number of reads per tested OTUs in individual samples). To account for deviance from a Gaussian error distribution, the significance of the GIT region effect was assessed based on permutations. In particular, observed deviance changes due to the elimination of the GIT region effect for the initial model were compared with the null distribution of deviance changes extracted from LMEs, where both the number of total and OTU-specific read counts were randomly resampled (10 000 permutations). The Qvalue method [80] was used to account for false discoveries due to multiple testing. The effect of a given OTU was considered to be significant if the permutation-based p value and associated qvalue were lower than 0.05. The abundance pattern of OTUs that were overrepresented in the oral cavity or faecal samples was visualized using a heatmap (function aheatmap from R package NMF).

## Results

**Fig 1:**
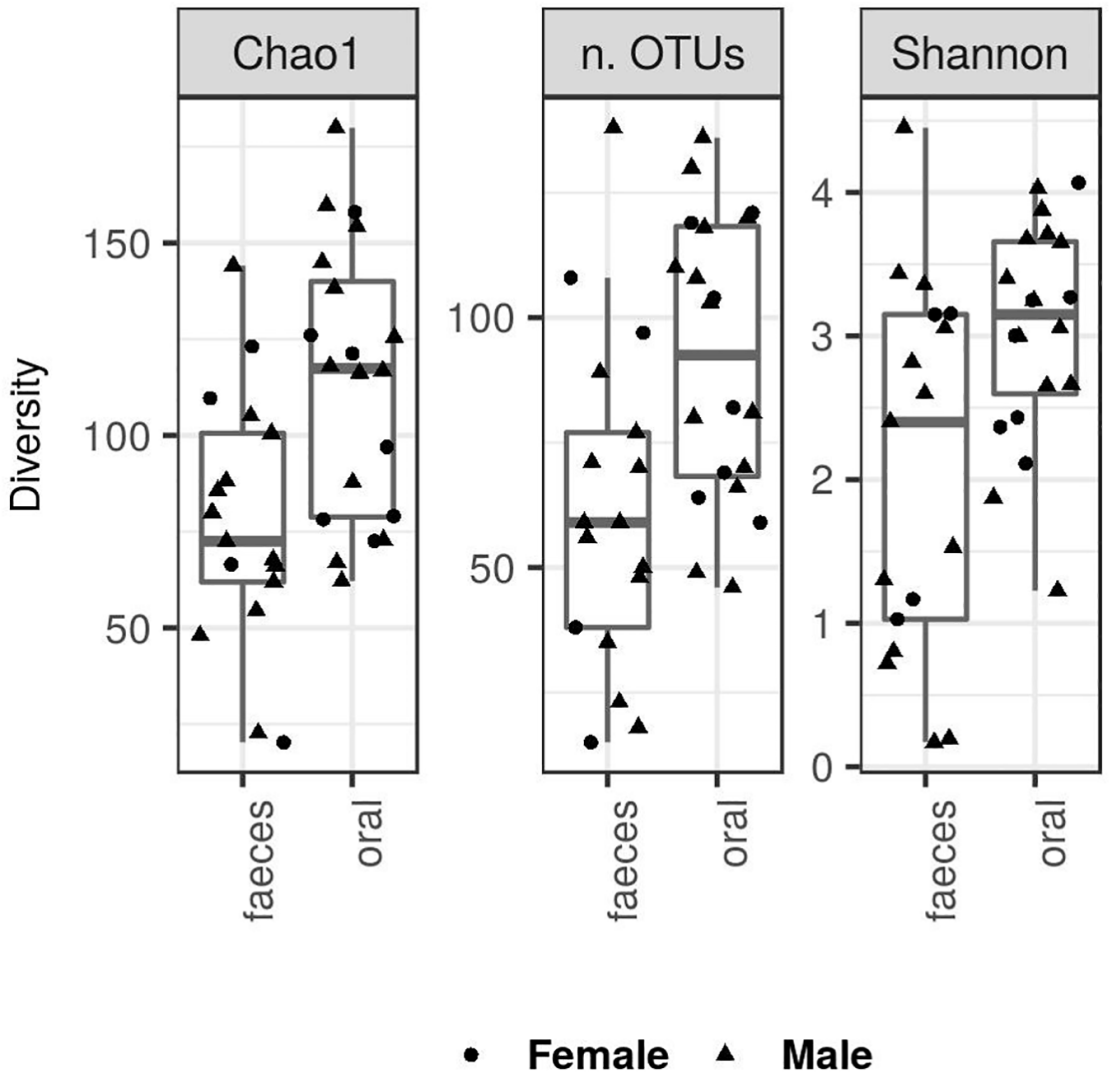
Diversity of the faecal and oral microbiota of the great tit. Alpha diversity was measured as Chao1, number of observed OTUs and Shannon diversity. To account for uneven sequencing depths, alpha diversities were calculated based on rarefied OTU tables.

**Fig 2:**
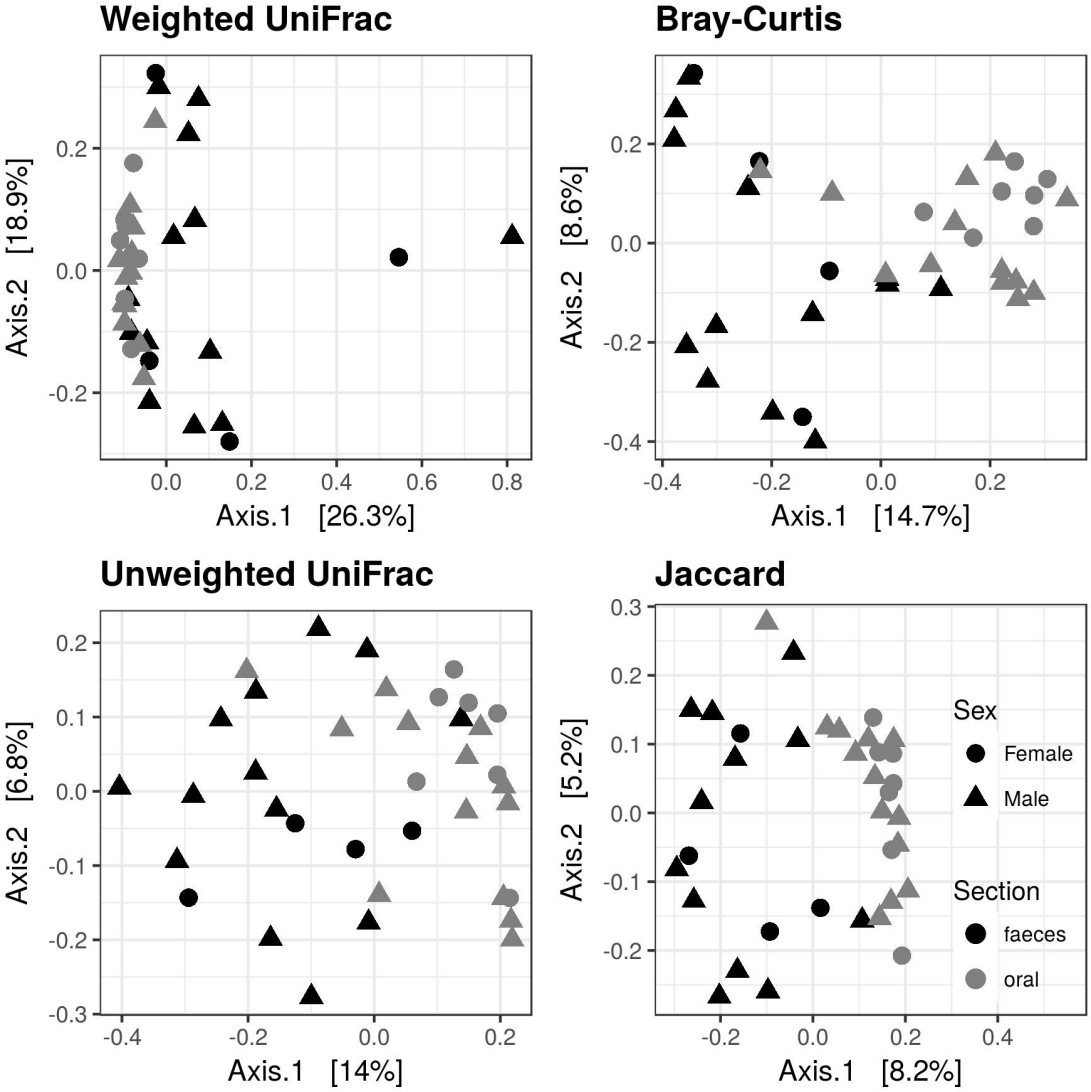
Differences in the composition between the oral and faecal microbiota of the great tit. PCoA was performed for four dissimilarity indexes. Sex is indicated by different plotting symbols.

**Fig 3:**
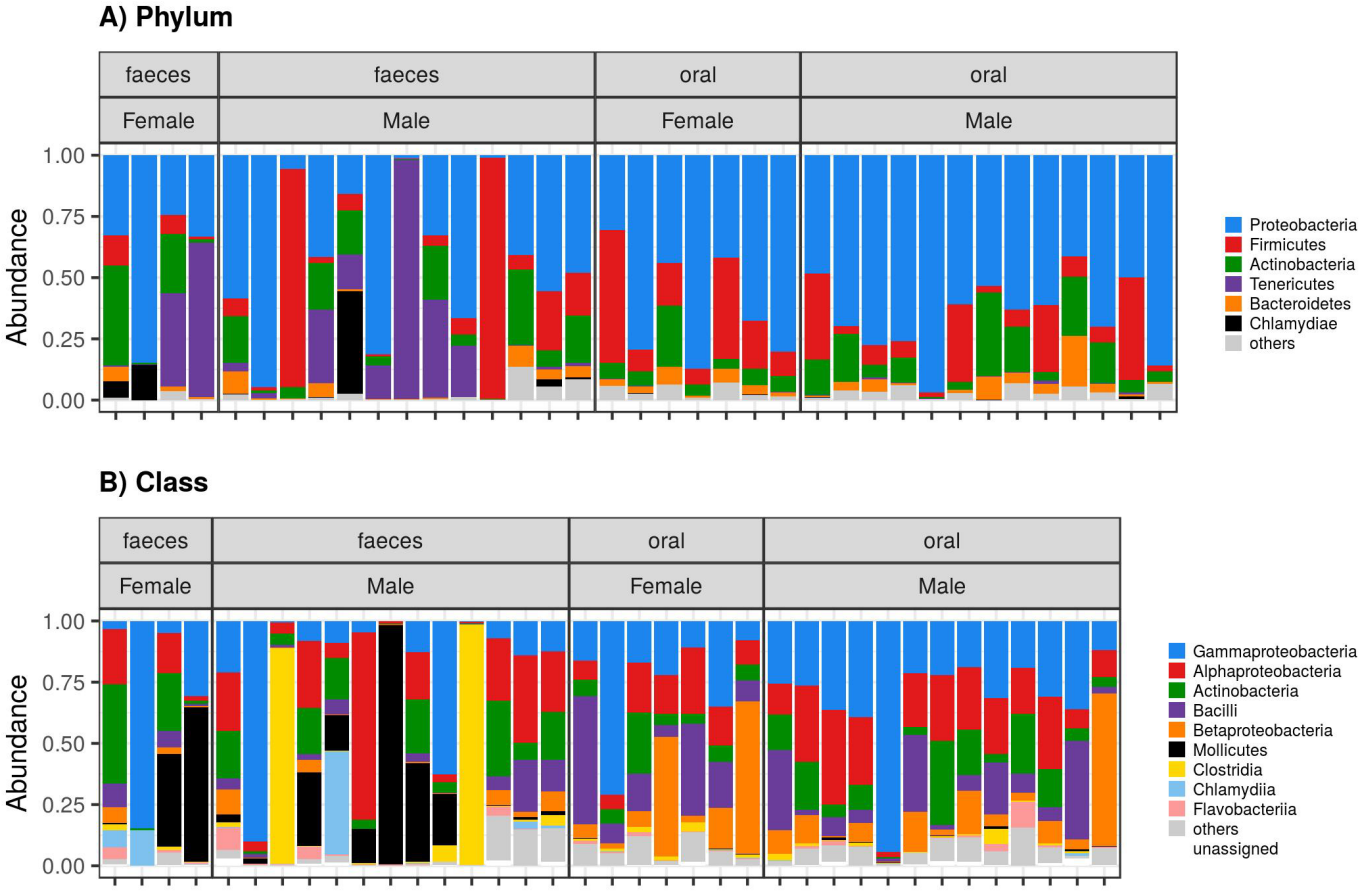
Barplots indicating oral and faecal microbiota composition of the great tit. Proportions of bacterial (*a*) phyla and (*b*) classes are shown.

Our dataset included 207 497 high-quality reads that were clustered into 1127 non-chloroplast OTUs. There was a significant decrease of alpha diversity in faecal compared to oral microbiota according to the observed number of OTUs and Shannon index, as well as according to Chao1 (Fig 1, Table 1). Only 384 (34%) OTUs were detected in both the oral and faecal microbiota, whereas 541 (48%) and 202 (18%) OTUs were detected exclusively in oral and faecal samples, respectively. Clear differences in the composition of oral vs. faecal microbiota were also revealed based on PCoA (Fig 2) and the associated adonis analyses (Table 2). In addition, the interindividual variation in the microbial composition, as assessed by betadisper analyses, was lower in oral compared to faecal samples for all types of community dissimilarities, but no significant difference was found in the unweighted UniFrac (Table 2). In line with these results, plots visualising the taxonomic composition on the Phylum and Class levels indicated differentiation in the microbial composition between faecal and oral samples as well as a higher interindividual variation of faecal microbiota (Fig 3, S2 Table). Gammaproteobacteria (genera *Diplorickettsia*, *Pseudomonas*, *Erwinia*, *Escherichia/Shigella*, *Serratia* and *Acinetobacter*), Alphaproteobacteria (genera *Methylobacterium*, *Rickettsia* and *Sphingomonas*) and Actinobacteria (genera *Corynebacterium* and *Pseudonocardia*) were the dominating bacterial classes of both the oral and faecal microbiota. However, the abundance of Bacilli (represented by genera *Staphylococcus* and *Lactobacillus*) and Betaproteobacteria (represented by genera *Methylobacillus*, *Comamonas* and *Herbaspirillum*) was increased in oral compared to faecal samples. At the same time, several faecal samples exhibited high abundances of Mollicutes (represented by genera *Ureaplasma* and *Mycoplasma*), Clostridia and Chlamydia, i.e. bacterial classes that were detected in very low abundances in oral samples.

**Table 1:** Diversity of the faecal and oral microbiota of the great tit.

	Oral mean ± SE	Fecal mean ± SE	χ2	P
**Chao1**	119.0186 ± 10.0396	78.4456 ± 9.1118	8.21050	0.00416
**Observed**	92.2500 ± 6.6153	62.2353 ± 8.1274	7.93768	0.00484
**Shannon**	3.0168 ± 0.1724	2.0839 ± 0.3136	7.05950	0.00788

Alpha diversity was measured as Chao1, number of observed OTUs and Shannon diversity. Significance was assessed based on LME. Mean ± SE for individual sample groups, LME based likelihood-ratio statistic associated probability values are shown.

**Table 2:** Differences in the composition between the oral and faecal microbiota of the great tit.

			composition				interindividual variation		
		Df	Mean Sum Sq.	F	R^2^	p	Mean Sum Sq.	F	p
**Weighted UniFrac**	GIT region	1	0.47575	4.09496	0.10474	0.00781	0.24614	20.64496	0.00006
	Residuals	35	0.11618				0.01192		
**Unweighed UniFrac**	GIT region	1	0.78810	3.58368	0.09288	0.00781	0.00204	0.92960	0.34158
	Residuals1	35	0.21991				0.00220		
**Bray Curtis**	GIT region	1	1.51171	4.31907	0.10985	0.00391	0.09235	14.12805	0.00062
	Residuals2	35	0.35001				0.00654		
**Jaccard**	GIT region	1	0.92409	2.56352	0.06825	0.00391	0.00884	9.14144	0.00465
	Residuals3	35	0.36048				0.00097		

Differences in composition were analysed using adonis, whereas differences in interindividual variation were assessed using betadisper. Both analyses were performed on four types of dissimilarity indexes.

**Fig 4:**
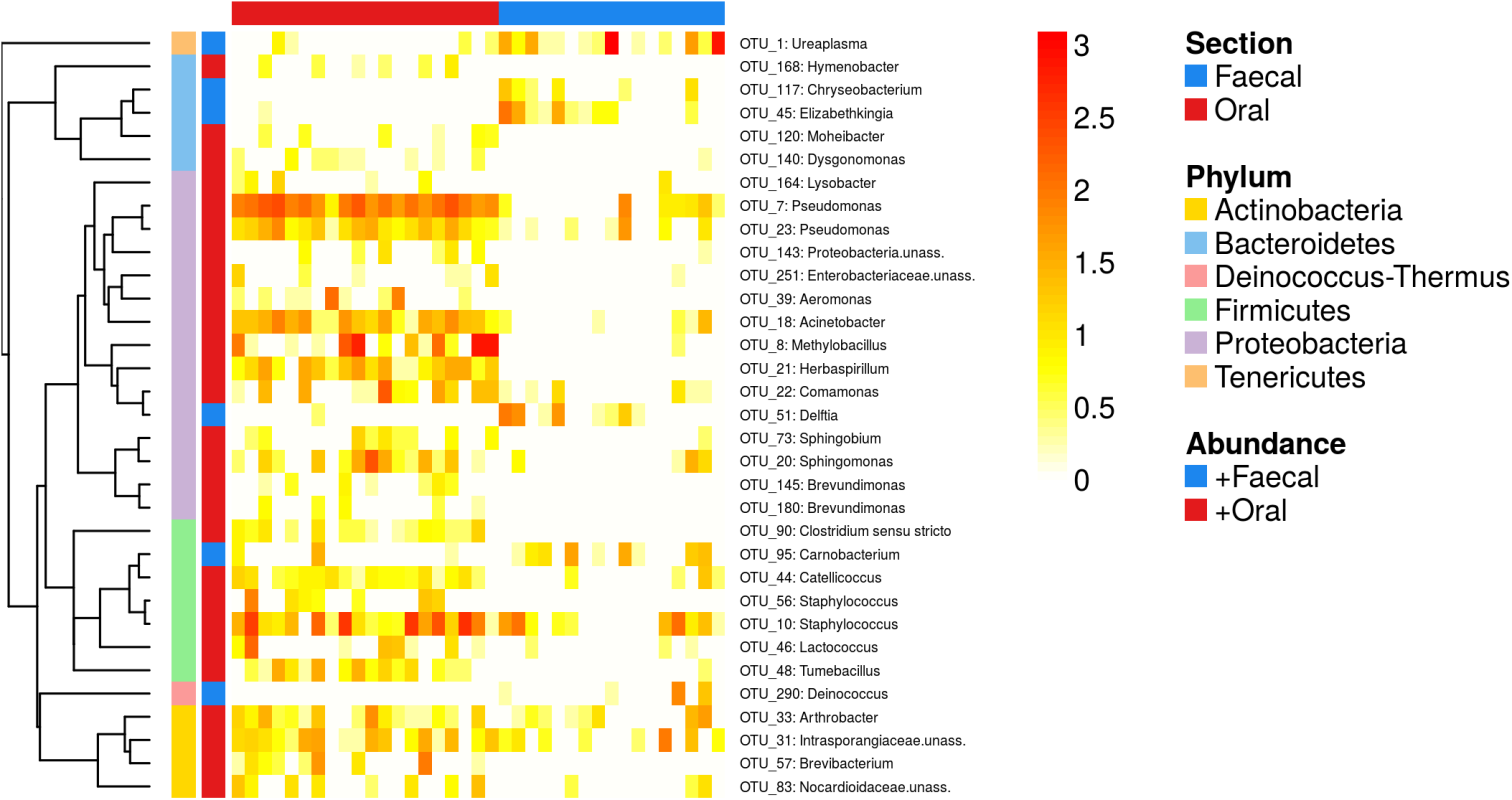
Heatmap for OTUs, whose abundance varied between the oral and faecal microbiota of the great tit. OTUs were identified according to permutations-based LMEs. Cell colours indicate OTU abundances in individual samples (log10 scaled values). Column annotations indicate GIT regions, whereas row annotations show Phylum-level assignations and if the OTU was overrepresented in oral or faecal samples (+oral or +faecal). OTUs are ordered according to their phylogeny (i.e. FastTree-based phylogeny for representative sequences).

OTU-level analyses identified 33 OTUs (represented by 35.8% reads) whose relative abundances differed between oral and faecal samples (Fig 4). OTUs corresponding to the genera *Ureaplasma* (phylum Tenericutes), *Delftia* (phylum Proteobacteria), *Carnobacterium* (phylum Firmicutes), *Deinococcus* (phylum Deinococcus-Thermus), *Chryseobacterium* and *Elizabethkingia* (both phylum Bacteroidetes) were more abundant in faecal samples. On the other hand, OTUs where the most striking relative abundance increase in oral compared to faecal samples was observed belonged to the genera *Pseudomonas*, *Acinetobacter*, *Methylobacillus*, *Herbaspirillum* (all from phylum Proteobacteria), *Catellicoccus*, *Staphylococcus*, *Tumebacillus* (phylum Firmicutes) and to *Arthrobacter*, *Brevibacterium* and the families Intrasporangiaceae and Nocardioidaceae (phylum Actinobacteria).

ANCOVA analyses did not reveal any effect of sex or scaled body mass index on the diversity of microbial communities associated with these two GIT regions (p < 0.3 in all cases). In addition, according to adonis analyses, there was no effect of these two variables on the composition of oral or faecal microbiota (p > 0.2, R^2^ < 0.02 in all cases).

Analyses on the subset of individuals with both oral and faecal microbiota sampled did not reveal any correlation of the microbial structure between these two GIT regions at the within-individual level. First, alpha diversity estimates for these two GIT regions were not correlated (range of Pearson's r = -0.22 ~ -0.26, p > 0.5 for all types of diversity indexes). Furthermore, we did not detect any within-individual correlation in microbial composition between faecal and oral samples (Mantel test: p > 0.4, range of cor. coefs. = -0.25 ~ 0.19 for all four community dissimilarity indexes). Finally, the correlation of relative abundances of individual OTUs between oral swabs and faeces was negligible (mean of Spearman correlation coefficient = 0.05185, interquartile range = -0.21600 ~ 0.32870).

## Discussion

Many previous studies that focused predominantly on captive bred mammals and humans have found pronounced differences in microbial structure between body sites as well as among different GIT compartments [37,81,82]. Our aim was to extend current knowledge on microbial divergence between GIT regions with data from the free-living population of a passerine bird, i.e. a taxonomic group that, to our knowledge, has not been studied in this context before. Consistent with previous research, we found pronounced differences between the proximal vs. distal GIT microbiota. According to our data, less than 30% of all OTUs were shared between these two GIT compartments in the great tit. Differences in terms of OTU absence vs. presence reflected pronounced variation in the relative abundances of bacterial taxa that were detected in oral vs. faecal microbiota. Compared to faecal microbiota, oral samples were characterized by a higher proportion of the classes Bacilli (phylum Firmicutes) and Betaproteobacteria (phylum Proteobacteria). In addition, bacteria from the phyla Chlamydiae and Tenericutes as well as from the class Clostridia (phylum Firmicutes) were nearly absent in the great tit oral microbiota.

Altogether, the composition of the great tit faecal microbiota was comparable with previous studies on other passerine birds [29,30,60,83–86], where OTUs from the phyla Proteobacteria, Firmicutes and Actinobacteria represented the dominant components. On the other hand, knowledge on the taxonomic content of the oral microbiota in birds is currently very limited. However, our data indicate that there is pronounced variation between the great tit and other avian hosts. For example, a dominance of *Lactobacilli* was detected in a recent study on the quail (*Coturnix japonica*) [87], while this bacterial genus constituted only a low proportion of the oral microbiota in our population. Similarly, abundances of *Haemophilus* and *Streptococcus* that dominated the oral microbiota of the kakapo (*Strigops habroptilus*) [88] were low in the great tit. Compared to mammalian oral microbiota, where bacteria from the phyla Bacteroidetes, Firmicutes and Proteobacteria typically dominate, the oral microbiota in our population was characterized by a low proportion of Bacteroidetes and increased abundances of Actinobacteria [40,43,82,89]. In addition, the diversity of the oral microbiota was significantly increased compared to the faecal microbiota in our population. As studies on other vertebrate species commonly report both higher [1,40,43,90] and lower [82,91] values of alpha diversity in oral vs. faecal microbiota, further research should focus on factors driving this variation.

At the OTU level, the faecal microbiota was characterized by increased abundances of *Ureaplasma*, *Deinococcus*, *Carnobacterium*, *Chryseobacterium*, *Delftia* and *Elizabethkingia* OTUs. *Ureaplasma* together with another Tenericutes OTU corresponding to the genus *Mycoplasma* that tended to be increased in the faecal microbiota as well (qvalue ~ 0.08), are common inhabitants of vertebrate gastrointestinal and urogenital tracts. Although these taxa are often asymptomatically present in birds, some of these species are involved in severe pathogenesis [92]. The *Deinococcus* OTU (phylum Deinococcus-Thermus) has previously been detected in several vertebrate species [85,93]; however, its effect on the host physiology is poorly known. The *Carnobacterium* OTU (phylum Firmicutes) is a lactic acid bacterium with putative probiotic properties providing protection against various bacterial pathogens [94,95]. On the other hand, *Chryseobacterium* and *Elizabethkingia* OTUs (both from the family Flavobacteriaceae) are related to several pathogenic species of human and other vertebrate taxa [96–98].

According to the OTU-level analyses, the oral microbiota was characterized by increased abundance of OTUs from genera that commonly colonize the oral cavity, skin or intestine of various vertebrate taxa (for example *Staphylococcus*, *Acinetobacter*, *Sphingomonas*, *Brevundimonas*, *Dysgonomonas*, *Hymenobacter*, *Sphingobium*). Many OTUs exhibiting higher abundances in the oral cavity compared to faeces can be involved in interactions with their host's immune system and physiology, or can shape the community composition via interactions with other members of the oral microbiota. This applies, for example, to four Actinobacterial OTUs (from the genus *Arthrobacter*, *Brevibacterium* and the family Intrasporangiaceae, Nocardioidaceae). Actinobacteria produce a wide variety of bacteriocines and other compounds suppressing the proliferation of bacterial competitors. Therefore, their presence in the oral cavity could be crucial for the defence against bacterial pathogens as well as for the maintenance of overall microbial structure [99]. In line with this possibility, *Arthrobacter* abundances in the lower intestine were positively associated with survival rates of passerine species closely related to the great tit [59]. Other bacteria associated with the oral cavity that may be involved in interactions with invading pathogens and other community members included *Lysobacter*, *Pseudomonas* and *Herbaspirillum*. *Lysobacter* can shape microbial community through the production of bacteriocines and the active predation of other bacteria [100,101]. *Pseudomonas* and *Herbaspirillum* produce extracellular siderophores, i.e. iron chelating compounds, which provide them a competitive advantage over other bacteria by reducing the availability of iron in the environment [102]. In addition, *Pseudomonas* cells secrete exopolysaccharides that make them a difficult target for the host immune system [103], and some *Pseudomonas* species can be pathogenic for birds [104–106]. It is also worth noting the abundance increase of two lactic acid bacteria in the oral cavity, *Catellicoccus* and *Lactococcus*, which can shape the oral community structure by the modulation of abiotic environmental conditions or by direct interactions with host immune system or other community members [107,108].

Even though the sample size was limited, we did not observe any correlation between oral vs. faecal microbiota at the within-individual level. Consequently, we propose that microbial communities associated with the proximal and distal GIT are shaped by independent mechanisms. These can theoretically include (1) host-intrinsic mechanisms such as effects of the immune system and other biotic and abiotic factors operating within the GIT, or (2) extrinsic sources of variation including pools of bacteria present in the diet and other environmental sources.

Relatively low interindividual variation of oral microbiota suggests either that environmental bacterial pools colonizing the oral cavity exhibit high homogeneity in space and time, or that there is low interindividual variation in host-specific mechanisms regulating oral microbiota. As previous research has found only limited effects of environmental bacteria on oral microbiota in non-avian vertebrates [43,50], and as environmental microbial consortia typically exhibit high variation [109–111], we favor the latter explanation. High interindividual variation of faecal microbiota suggests that host-intrinsic factors driving its composition differ markedly among hosts. However, as the passage of food through the passerine gut is extremely fast [112,113] and thus the decomposition of bacteria from the external environment is probably not as effective as in mammals, we cannot exclude the possibility that the variation of faecal microbiota is also driven to certain extent by bacteria that get into the GIT with food. As knowledge of the factors driving within-species variability in the avian GIT microbiota remains limited [59,61,85], further research and specifically designed experiments are required to untangle the relative contribution of transient environmental bacteria to microbial composition in different GIT regions of passerines and other vertebrate taxa.

In conclusion, our study is the first to characterize the oral microbial structure and compared it with the faecal microbiota in a free-living bird population. Our results show that the oral and faecal microbiota of passerines represent two distinct bacterial consortia that exhibit marked differences at all levels of community structure, and that the interindividual variation of these communities is likely to be shaped by independent mechanisms. We propose that aside from the effect of environmental bacteria, the structure of both the faecal and oral microbiota is driven to a large extent by mutual interactions among community members or by the host vs. microbiota interactions including immunity. Consequently, given the putative effects of these two microbial communities on the host's heath status, further research focusing on the microbiota in wild vertebrate populations may benefit from simultaneous sampling of these two communities.

## Supporting information

S1 Table Detail listing of great tit samples including the GIT region (faecal or oral), individual identity (ID), accession number, sex and body weight. (XLSX)

S2 Table Taxonomic composition of the great tit GIT microbiota and its variation between oral and faecal samples. (XLSX)
